# Single-Nucleotide Polymorphisms (SNPs) in Vitamin D Physiology Genes May Modulate Serum 25(OH)D Levels in Well-Trained CrossFit^®^ Athletes, Which May Be Associated with Performance Outcomes

**DOI:** 10.3390/ijms26125602

**Published:** 2025-06-11

**Authors:** Diego Fernández-Lázaro, Juan Mielgo-Ayuso, Jesús Seco-Calvo, Eduardo Gutiérrez-Abejón, Enrique Roche, Manuel Garrosa

**Affiliations:** 1 Area of Histology, Faculty of Medicine and INCYL, University of Valladolid, 47005 Valladolid, Spain; manuel.garrosa@uva.es; 2 Neurobiology Research Group, Faculty of Medicine, University of Valladolid, 47005 Valladolid, Spain; 3 Research Group “Nutrition and Physical Activity”, Spanish Nutrition Society “SEÑ”, 28010 Madrid, Spain; jfmielgo@ubu.es (J.M.-A.); eroche@umh.es (E.R.); 4 Department of Health Sciences, Faculty of Health Sciences, University of Burgos, 09001 Burgos, Spain; 5 Institute of Biomedicine (IBIOMED), University of León, 27071 León, Spain; jesus.seco@unileon.es; 6 Pharmacological Big Data Laboratory, Department of Cell Biology, Genetics, Histology and Pharmacology, Faculty of Medicine, University of Valladolid, 47003 Valladolid, Spain; egutierreza@saludcastillayleon.es; 7 Pharmacy Directorate, Castilla y Leon Health Council, 47007 Valladolid, Spain; 8 Atención Primaria, Área de Salud de Valladolid Este, 47010 Valladolid, Spain; 9 Department of Applied Biology-Nutrition, Institute of Bioengineering, Miguel Hernandez University, 03220 Elche, Spain; 10 Alicante Institute for Health and Biomedical Research (ISABIAL), 03010 Alicante, Spain; 11 CIBER Fisiopatología de la Obesidad y Nutrición (CIBEROBN), Instituto de Salud Carlos III (ISCIII), 28029 Madrid, Spain

**Keywords:** 25-OH vitamin D, nutrigenetic, SNPs, sports performance, CrossFit^®^

## Abstract

Vitamin D is a key micronutrient in the function of the skeletomuscular system. Athletes are at increased risk of developing vitamin D deficiency during the execution of very demanding disciplines such as CrossFit^®^. Single-nucleotide polymorphisms (SNPs) may influence circulating 25-hydroxy-vitamin D (25(OH)D) levels. An observational, longitudinal pilot study was conducted with 50 trained males according to specific inclusion criteria. Blood samples were obtained to determine *25(OH)*D, *vitamin D-binding protein* (*VDBP*), *vitamin D-receptor* (*VDR*)circulating levels, and the presence of SNPs after DNA isolation and genotyping: rs10741657 to *CYP2R1*, rs2282679 to *GC* and rs2228570 to *VDR* genes. Significant differences (*p* < 0.05) in 25(OH)D concentration were determined between the biallelic combinations of rs228679 (*GC*) and rs228570 (*VDR*). The VDBP and VDR proteins did not show different levels in the case of the rs10741657 (*CYP2R1*) alleles. Statistically significant weak positive correlations (*p* < 0.05) were observed between 25(OH)D and AA-alleles of the *CYP2R1* and *VDR* genes, and TT-alleles of the *GC* gene. Additionally, *AA* (rs10741657 and rs2228570) and *TT* (rs2282679) have a probability between 2 and 4 of having major effects on the concentration of 25(OH)D. Conversely, *GG* alleles present a probability of suboptimal values of 25(OH)D of 69%, 34%, and 24% for *VDR*, *GC*, and *CYP2R1*, respectively, showing a strong moderate positive correlation (r = 0.41) between the degrees of sports performance and 25(OH)D plasma levels. *CYP2R1* (rs10741657), *GC* (rs2282679), and *VDR* (rs2228570) affect the concentration of serum 25(OH)D, as an indicator of vitamin D status and play a critical role in the sports performance of CrossFit^®^ practitioners.

## 1. Introduction

Several reports confirm that optimal levels of serum 25-hydroxy-vitamin D (25(OH)D) correlate positively with sports performance, including strength and power, running, endurance, and aerobic abilities [1,2,3,4]. In addition to its well-established role in bone metabolism, vitamin D is a key micronutrient in the function of the nervous, immune, and muscular systems [5]. Athletes who present low levels of circulating vitamin D and perform very demanding activities (high-intensity and long duration) are at high risk of musculoskeletal injuries, immunosuppression, or arthritis [6,7]. One of the factors for the decrease in athletic performance seems to be an inadequate recovery [8]. In this line, the attenuation of muscular weakness after very demanding exercise requires maintaining optimal circulating levels of vitamin D [9], which can be achieved through adequate supplementation. Altogether, this is the reason why 25(OH)D deficiency is estimated at 20 ng/mL (50 nM) for sport practitioners [6] compared to 10 ng/mL (25 nM) for the non-athletic population [10]. Several factors predispose vitamin D deficiency, such as changes in body composition, dark skin, young age, frequent indoor sports activities, and not meeting sports dietary recommendations, among others [11]. Nevertheless, the most determinant factor is intensity of sport actions. In this line, athletes are at increased risk of developing 25(OH)D deficiency during the execution of very demanding disciplines.

CrossFit^®^ is a branded fitness regimen that incorporates high-intensity interval training (HIIT), and it is a specific type of functional fitness training, focusing on a variety of movements at high intensity [12]. In the context of CrossFit^®^, “Workouts of the Day” (WODs) are standardised, varied, and high-intensity routines that are core to the program. These routines are designed to improve overall fitness by incorporating functional movements and HIIT [12,13]. WODs are exercises with functional movements to promote muscular strength and cardiorespiratory fitness [14], executed at high speed during specific periods of time, with little or no rest between routines. WOD actions require coordination, agility, and precision [15]. This overload situation can lead to early fatigue, increased oxidative stress, extreme musculoskeletal damage, and an acute decreased performance from overtraining [16]. The extreme physical and energetic demands of CrossFit^®^ training demand particular nutritional requirements [17,18]. Insufficient nutrient intake could favour the appearance of the syndrome of “Relative Energy Deficiency in Sport (RED-S)” [19], which is associated with low levels of vitamin D [20]. RED-S occurs due to the low availability of energy substrates to carry out the very demanding physical performance during exercise execution, as it is the case of CrossFit^®^. RED-S affects all types of athletes regardless of gender or sport level [21]. Therefore, nutritional practices must be implemented, allowing to cover the specific requirements of CrossFit^®^ practitioners. These include ketogenic diet [22,23], optimal protein [24] and carbohydrate [25] intakes before and after training or the use of some ergogenic supplements such as caffeine [26], sodium bicarbonate [27], nitrate [28], β-hydroxy-β-methylbutyrate [29] and herbal supplements such as green tea [30] or *Tribulus terrestris* L. extracts [16,31].

Therefore, CrossFit^®^ practitioners seem to expose their bodies to physical limits. To achieve maximum sports performance requires a correct nutritional planning and supplementation with nutrients that do not reach optimal levels only with diet. In this context, vitamin D is of particular importance because of its key role in the nervous, immune, muscular, and skeletal systems [5,8]. For all these reasons, vitamin D seems to acquire a more relevant role in CrossFit^®^ practitioners by preserving bone, immune, and muscle health [8].

Vitamin D supplements correlate positively with improvements in the strength of lower extremities assessed by vertical jump in soccer players [3]; meanwhile, supplements have no effect in isokinetic actions in the same body segments and sport discipline [32]. These discrepancies could be due to genetic determinants that may influence circulating 25(OH)D levels [33]. In this line, single-nucleotide polymorphisms (SNPs) have been reported to influence nutrient metabolism, including vitamin D status [33]. SNPs appear to modify individual responses related to athlete health and performance [34]. In addition, certain SNPs could modulate (increase or decrease) the concentration of bioactive nutrients in plasma [35], and contribute to regulating muscular strength and mass, and hence sports performance status [7]. Currently, research is trying to identify multifactorial genetic factors, which involve all the biological processes (metabolism, transport, and interaction with the receptor) relationship with the functionality of vitamin D, through the evaluation of the genome using SNPs [33].

Regarding vitamin D, SNPs in the *CYP2R1* gene have an impact on vitamin D metabolism. *CYP2R1* codes for the hepatic 25-hydroxylase of the cytochrome P450 family [36], responsible for the first hydroxylation in the metabolic pathway to the active form of vitamin D, which is a critical step in the functional transformation of the vitamin [37]. Lower levels of active vitamin D as a result of SNPs in *CYP2R1* could potentially lead to decreased muscle strength and function [33], impacting sports performance, particularly in activities requiring strength and power [38]. In addition, SNPs in the *GC* gene coding for vitamin D-binding protein (*VDBP*) influence vitamin D transport. *VDBP* is a protein belonging to the albumin family, particularly *VDBP* is the main carrier of vitamin D but also of its metabolites, 25(OH)D, 1,25-dihydroxy vitamin D (1,25(OH)_2_D) and also 24,25-dihydroxy vitamin D (24,25(OH)_2_D) in the blood. *VDBP* transports them to regulate the access of all vitamin D metabolites to cells and tissues for binding to the receptor [39,40]. *VDBP* has a single binding site for all vitamin D metabolites and has a high affinity for 25(OH)D, thus creating a large pool of circulating 25(OH)D, which prevents rapid vitamin D deficiency when the supply of vitamin D is compromised [41], including athletes with high physical demands (present report). Furthermore, other metabolic functions of *VDBP* that could influence muscle performance have been reported, such as the modulation of inflammatory processes and innate immunity, and the regulation of bone metabolism [42].

Finally, SNPs in the vitamin D-receptor (*VDR*) gene influence vitamin D biological activity in many tissues, being an aspect of particular interest in sports performance [43,44,45,46,47]. The rs2228570 (*FokI*) is the only polymorphism perturbing the length and functionality of the VDR protein [48]. VDR is present in muscle cells; it is in this tissue that VDR regulates the cellular proliferation and differentiation of muscle cells, as well as the transport of calcium and phosphate to intracellular compartments [49]. VDR functions in tissue regeneration, by regulating protein synthesis, and muscle function, by participating in muscle contraction and the transmission of nerve impulses (due to the redistribution of calcium and phosphate) [50]. Furthermore, deficient (less than 10 ng/mL) or toxic (more than 150 mg) 25(OH)D levels inactivate the biologically active form of VDR [51]. Altogether, VDR has a great interest in athletics.

In view of the foregoing information (link of vitamin D deficiency with suboptimal performance, recovery, immune dysregulation, and high risk of muscle injuries), we conducted a pilot study to evaluate a possible connection between the presence of certain SNPs in *CYP2R1* (rs10741657), *GC* (rs2282679) and muscle *VDR* (rs2228570) genes, with serum 25(OH)D concentrations and the degree of WOD performance in trained CrossFit^®^ practitioners. Knowing these relationships could be instrumental for personalised vitamin D supplementation and training/recovery strategies.

## 2. Results

### 2.1. CrossFit^®^ Practitioners’ Characteristics

**Figure 1:**
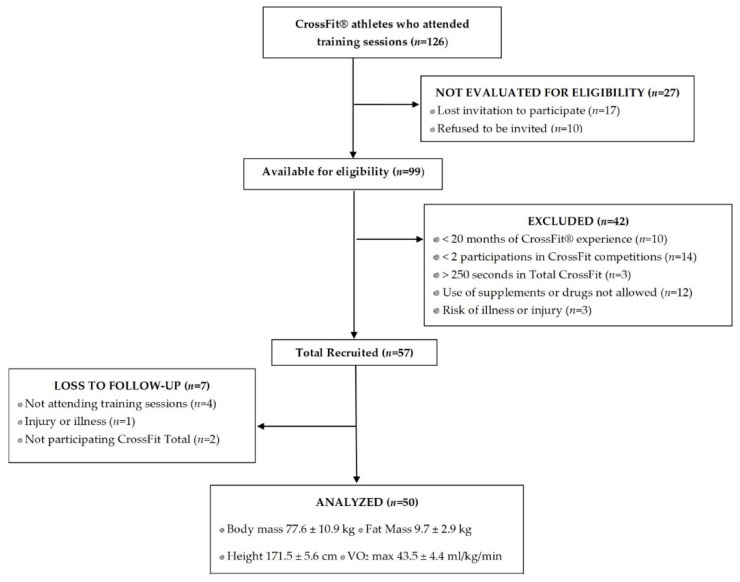
STROBE flow diagram for recruitment.

The participant flow diagram is depicted in Figure 1. Characteristics of participants are indicated in Table 1. All participants were male. Information regarding ethnicity was obtained in order to discard vitamin D variations due to the ethnic origin of participants. Although this is a topic of intense debate, it seems that people with dark skin have a risk of a deficient synthesis of vitamin D [52]. In this line, all participants were Caucasian males and living in very close locations in Spain, undergoing similar sun exposure according to their lifestyle. On the other hand, anthropometric parameters describe participants with optimal fat mass and displaying normal weight. The VO_2_max values are included into the range observed for the general population: 40–50 mL/kg/min. Participants had more than 20 months of experience in CrossFit^®^ training and performed Fran WOD in less than 250 s. Among the 126 participants who attended training sessions, 27 athletes were not screened for eligibility due to missed invitations to participate (n = 17) or declined the invitation (n = 10). A total of 99 CrossFit^®^ athletes were eligible and invited to participate in the study; however, 42 (42.4%) participants were excluded for not meeting the inclusion criteria, and 7 (7.1%) athletes were excluded for loss to follow-up. Therefore, 50 participants met the inclusion criteria and were included in the final study sample, as shown in the diagram in Figure 1.

### 2.2. CrossFit^®^ Practitioner’s Dietary Assessment

Table 2 shows the energy and micronutrient intake of CrossFit^®^ participants. Regarding macronutrients, intakes were considered appropriate for intervallic sport disciplines: 4–6 g of carbohydrates/Kg of body weight, 1–1.5 g of lipids/Kg of body weight and 1.4–1.8 g of protein/Kg of body weight [53]. Although this an observational study, we corrected minimal differences in some participants at the beginning of the study, in order to have a more homogeneous diet in the group of participants. Regarding minerals and vitamins, they were into the recommended dietary allowances [54].

### 2.3. Circulating 25-Hydroxy-Vitamin D Plasma Levels by Ranges

Circulating 25(OH)D levels classified according to sports practice [55] are indicated in Table 3. The age cut-points used were based on the capacity of participants to perform CrossFit^®^ competition, according to age cut-points corresponding to the “Absolute or Elite” category (18–35 years) and 35 years for the “Master” category, according to Competition Rulebook CrossFit^®^ [55]. Vitamin D deficiency can begin to be observed starting at age 35, especially in those with risk factors such as low sun exposure, use of sunscreen, and diets low in vitamin D [56]. Nevertheless, participants with deficient or high levels (predisposing to toxicity) of 25(OH)D were not noticed. On the other hand, 68% of participants displayed sufficient levels of 25(OH)D, and 16% presented insufficient levels. Only 16% of participants showed optimum circulating levels of 25(OH)D [6]. In this context, similar percentage (around 16%) of young as well as older individuals (more than 35 years old) displayed optimal levels of circulating 25(OH)D. However, insufficient levels of 25(OH)D were two times more abundant in old CrossFit^®^ practitioners, and sufficient levels were more abundant in old participants. Altogether, age seems to be a determinant variable to condition circulating 25(OH)D levels, but this is still a matter of scientific debate [57]. Nevertheless, and due to the low n, studies with more participants are necessary to decipher the role of age in 25(OH)D circulating levels in CrossFit^®^ practitioners.

### 2.4. Circulating of 25-Hydroxy Vitamin D, Vitamin D Binding Protein and Vitamin D Receptor Protein Plasma Levels

Table 4 reports circulating 25(OH)D levels present in CrossFit^®^ practitioners with different SNPs. Significant differences (*p* < 0.05) were observed in plasma concentration of 25(OH)D for participants carrying the SNP r2282679 in the *GC* gene, between alleles carrying the GG and GT genotypes with respect to the homozygous biallele TT. Individuals carrying the 2 allelic variants (GT and GG) in *GC* gene displayed insufficient 25(OH)D levels compared to the corresponding homozygous biallele TT that displayed optimal levels of circulating 25(OH)D. In addition, individuals carrying the homozygous biallele GG compared to AA for SNP r2228570 in the VDR gene showed significant differences (*p* < 0.05) in circulating 25(OH)D. Individuals with this allelic variant in *VDR* gene displayed as well significant insufficient 25(OH)D levels compared to the corresponding homozygous biallele AA that presented sufficient levels. The GA variant for *VDR* gene displayed insufficient levels of 25(OH)D, but differences compared to the AA variant were not significant (Table 4). Individuals with the biallelic variants (GA and GG) in *CYP2R1* gene presented insufficient circulating levels of 25(OH)D, but with no significant differences respect to participants carrying the corresponding homozygous AA biallele (Table 4). Altogether, individuals with the homozygous bialleles (AA, TT and AA) in the three genes (*CYP2R1*, *GC* and *VDR*, respectively) present sufficient–optimal levels of 25(OH)D (Table 2). However, practitioners carrying the rest of the bialleles displayed insufficient circulating levels of 25(OH)D. Meanwhile, in the case of GA and GG for *CYP2R1*, the differences with respect to the AA allele were almost significant. In addition, Table 2 shows the plasma levels of VDBP and VDR proteins. A significant difference (*p* < 0.05) was observed for the circulating levels of VDBP for GG allele, being less abundant than in the case of TT and GT alleles. Moreover, a significant difference (*p* < 0.05) was observed for plasmatic levels of VDR protein for GG allele, being less abundant than in the case of AA and GA alleles. VDBP and VDR protein do not display different circulating levels in the case of the *CYP2R1* alleles.

### 2.5. Genotyping and Phenotyping Frequencies

CrossFit^®^ athletes were genotyped for variants in *GC*, *CYP2R1*, and *VDR*, genes (Table 5). The allele frequencies of the three SNPs assessed in this study were in Hardy–Weinberg equilibrium (HWE) and are reported in Table 5. The frequency of the AA genotype in our group of CrossFit^®^ athletes was 17% and 42% for rs10741657 and rs2228570, respectively. The frequency of the TT genotype of rs228679 was 38%. The three SNPs (rs10741657, rs2282679, rs2228570) showed a major allele frequency (MAFr) like other studies with participants from European [58,59,60] or USA [40] populations. In this way, MAFr for rs10741657 was 59% for our CrossFit^®^ athletes vs. 66.8% [60], and 58% [58] for European participants, and 58% [40] for USA participants The SNP rs2282679 was 60% for our CrossFit^®^ athletes vs. 71.4% [60], and 70% [58] for European participants, and 67 [40] for USA participants. The SNP 2228570 was 60% for our CrossFit^®^ athletes vs. 62% [59], and 65% [58] for European participants and, 64% [40] for USA participants.

### 2.6. Correlations Between 25-Hydroxy Vitamin D and Single-Nucleotide Polymorphisms of the CYP2R1, GC and VDR Genes

In order to support these observations, we analysed possible correlations between the indicated SNPs and 25(OH)D circulating levels. A significant (*p* < 0.05) mild negative correlation [r = (−0.30) − (−0.45)] was found between the SNPs of the homozygous biallelic variants (GG) for *CYP2R1*, *GC* and *VDR* genes and 25(OH)D plasma concentration (insufficient circulating levels) (Table 6). However, the homozygous reference biallele AA for *CYP2R1* and *VDR* genes and TT for *GC* gene showed significant (*p* < 0.05) weak positive correlation (r = 0.15–0.30) with 25(OH)D circulating levels (sufficient-optimal levels) (Table 6). No significant correlation was found for the rest of biallelic variants in the SNPs of the studied genes (Table 6). Altogether, these data suggest that the presence of biallele GG for the three genes seems to be the only biallelic variant that correlates slightly with insufficient plasma levels of 25(OH)D.

### 2.7. Single-Nucleotide Polymorphisms of the CYP2R1, GC and VDR Genes Associated with 25-Hydroxy Vitamin D Plasma Level

To support this observation, a diagnostic/predictor model of the plasma concentration of 25(OH)D in subjects carrying the studied SNPs in *CYP2R1*, *GC* and *VDR* genes was generated after a multivariate logistic regression analysis. Individuals carrying an AA homozygous biallelic genotype (rs10741657 in *CYP2R1* and rs228570 in *VDR* genes) and TT (rs2282679 in *GC* gene) could be more prone (Odds Ratio = 2–3) to have sufficient/optimal levels of circulating 25(OH)D (Table 7). On the other hand, the multivariate logistic regression analysis shows that individuals carrying the homozygous biallele GG in *CYP2R1*, *GC* and *VDR* genes were not associated with sufficient/optimal circulating concentrations of 25(OH)D, being more relevant in *VDR* rs228570 (odds ratio = 0.31) (Table 7). These variants seem to predict insufficient levels of circulating 25(OH)D, confirming the correlation analysis presented in Table 6. In addition, correct body mass index, VO_2_max and young age are predictors of circulating sufficient/optimal levels of 25(OH)D (Table 4). However, they were not stronger predictors than the reference allelic variants, AA in *CYP2R1* and *VDR* genes, and TT in *GC* gene (Table 7).

### 2.8. CYP2R1, GC and VDR Gene Polymorphism; 25-Hydroxy Vitamin D Plasma Level; and Sports Performance Levels in the CrossFit^®^ Total

**Figure 2:**
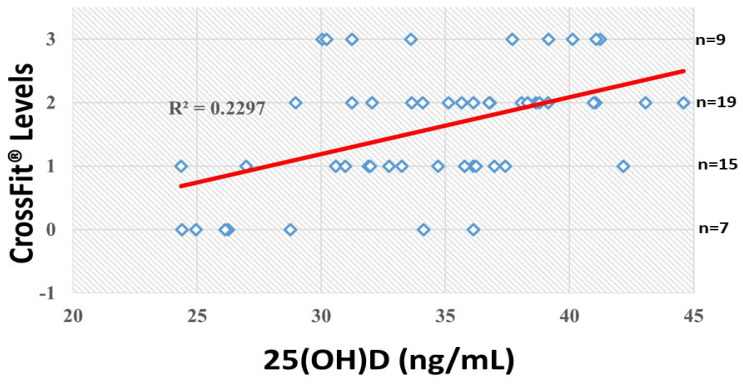
Correlation (R^2^) of performance levels by CrossFit^®^ Total and 25-hydroxy vitamin D (25(OH)D) plasma levels. The right column indicates the number (n) of subjects in each CrossFit^®^ level.

Altogether, the presented data indicate that certain SNPs in particular in genes coding for proteins involved in vitamin D function can condition 25(OH)D levels and thereby create optimal performance or subsequent recovery post-exercise. In this line, vitamin D deficiency is estimated at 20 ng/mL (50 nM) for sports practitioners [6], two times more than in no active population [10]. In the present report, deficiency cases were not detected (Table 3). However, 16% of practitioners displayed insufficiency—in other words, close to deficiency 25(OH) levels (Table 3). For this reason, they could be candidates for personalised supplementation. According to the presented data, the criteria to consider for particular supplementation protocols in the studied population could include the presence of particular SNPs and CrossFit^®^ level, evaluated by CrossFit^®^ Total. The SNP distribution in practitioners from the present study with different CrossFit^®^ levels is indicated in Table 8. Eight, 9 and 11 individuals carry the GG genotype in *CYP2R1*, *GC* and *VDR* genes, respectively. This particular biallele is observed in one competitor for *CYP2R1* gene, in three competitors for *GC* gene and one competitor for the *VDR* gene. The competitor is the category with maximal demanding performance. Competitors carrying this biallele display suboptimal (insufficient-sufficient) levels of circulating 25(OH)D and could need a particular vitamin D supplementation due to the more demanding efforts performed in the competition level. In the same line, the 7, 6 and 10 beginner/intermediate individuals carrying the GG genotype for *CYP2R1*, *GC* and *VDR* genes, respectively, would likely need vitamin D supplementation to progress to more demanding levels, such as competitors. We are considering these intervention hypotheses for future research. In this context, Figure 2 indicates that eight individuals (five in level 0, two in level 1 and one in level 2) could be candidates for personalised supplementation due to the insufficient circulating levels of 25(OH)D.

## 3. Discussion

Vitamin D is relevant for health status and plays key functions in sports performance and post-exercise recovery. In this context, optimal vitamin D circulating concentrations are associated with anti-inflammatory, antioxidant and immune actions in muscle cells with particular interest in sport performance [6,7,43]. The pathways described to explain the action of vitamin D in the modulation of skeletal muscle function can be identified as genomic and non-genomic [61]. In the same way and as well as nuclear steroids [62], the genomic actions of vitamin D are performed through binding to nuclear receptors. VDR is included in this family of proteins being expressed in muscle cells [63,64,65,66]. The steps prior to the binding of 1,25(OH)_2_D (calcitriol) to VDR are considered non-genomic and include the hydroxylation of 25(OH)D by *CYP2R1* gene product [36] and the transport to the target muscle cells, by VDBP [39,40].

Starting with the non-genomic pathways, hydroxylation of 25(OH)D by CYP2R1 to1,25(OH)D is a previous step prior binding to VDBP for plasmatic transport [39,40] and to VDR [36] to achieve the genomic actions. The identified SNPs in *CYP2R1* and *GC* genes substantially affect vitamin D status [34]. The GG genotype of *CYP2R1* (rs10741657) and GG and GT bialleles in *GC* gene (rs2282679) were significantly associated with suboptimal 25(OH)D levels [39,40]. We observed similar results, although the 25(OH)D circulating levels of SNPs in the *CYP2R1* gene were not significant compared to the circulating levels (sufficient-optimal) of participants carrying biallele AA (Table 4). Nevertheless, a modest correlation was observed for suboptimal 25(OH)D plasma concentration and presence of biallele GG for *CYP2R1* gene (Table 6). Moreover, and consistent with these studies [39,40], CrossFit^®^ practitioners of the present report carrying the A allele for *CYP2R1* gene and the T allele for *GC* gene are prone to displaying 2–3 times higher levels of plasma 1,25(OH)_2_D. In fact, we found that 16 and 14 “competitive” sports grade athletes carried the A allele (rs10741657) and the T allele (rs2282679), respectively (Table 8). In addition, other metabolic functions have been reported for VDBP that could influence muscle performance likely through the modulation of inflammatory processes [67]. In our study, individuals carrying the GG biallele for *GC* gene display lower circulating levels of VDBP. Therefore, VDBP primarily binds (85–90%) to 25(OH)D and transports it, so a lower serum concentration of VDBP means that less is bound, resulting in freer 25(OH)D. This situation allows free 25(OH)D (or increases its availability for cellular diffusion) to more easily enter cells by passive diffusion through the extracellular fluid [68]. However, passive diffusion, while facilitating entry, does not guarantee it. It depends on factors such as the size and polarity of the molecule, as well as the temperature and concentration gradient. Although free 25(OH)D is a small, nonpolar molecule, which favours diffusion, it is not guaranteed to enter all cells, especially if there is no favourable concentration gradient [69]. In addition, affected individuals seem to be likely exposed to suffer post-exercise inflammation due to a lower binding of VDBP to macrophage activating factor (MAF) [70], evading the modulation of post-exercise inflammation and compromising subsequent recovery. In this context, VDBP participates in actin removal, released after trauma by damaged muscle cells. Actin binds to coagulation factors, causing intravascular coagulation and subsequent muscle failure [71,72]. Therefore, the vitamin D–VDBP complex exerts post-exercise recovering actions, including binding to released actin microfilaments, resolving tissue injury and modulating inflammatory processes [67,73]. Altogether, optimal recovery is essential after demanding efforts in CrossFit^®^ practitioners. Nevertheless, this is an interesting hypothesis for future research.

Non-genomic pathways favour direct actions of vitamin D in muscle function, such as calcium transport to the sarcoplasmic reticulum, allowing muscle contraction and improving skeletal muscle functionality [44,74,75]. This is a key point for optimal performance in CrossFit^®^ practitioners. In this context, optimal vitamin D circulating levels are associated with neuromuscular performance [6,43,75], showing a positive association with muscle strength and recovery [6,75], particularly in the elderly [33,76]. These observations are consistent with the results reported in the present study with CrossFit^®^ practitioners regarding the strength performance test using the sum of repetition maximum (RM) on 3 different muscle groups (shoulder press, back squat, and deadlift) (Table 8). In fact, the main part of CrossFit^®^ competitors possesses the bialleles that correlate with the optimal concentrations of circulating 25(OH)D levels (Table 8). Specifically, the bialleles AA (*CYPR2R1*, *VDR*) and TT (*GC*) showed levels of 25(OH)D ≥ 30 ng/mL (Table 6), improving muscle strength and performance [6,75,77].

On the other hand, the biologically active form of vitamin D (1,25(OH)_2_D) binds to VDR to exert genomic downstream effects [73,78,79]. In the genomic pathway, known as well as slow or nuclear pathway, 1,25(OH)_2_D-VDR complex binds to vitamin D responsive elements (VDRE) located in promoters of specific genes, regulating its expression [31]. These genes code for specific proteins involved in key essential muscle functions, such as satellite cell (SC) proliferation and differentiation [80,81], inhibiting myostatin (GDF-8) (a negative regulator of muscle proliferation) [33], and concomitantly stimulating insulin-like growth factor-1 (IGF-1) (a main muscle anabolic factor) [82]. Altogether, these actions stimulate skeletal muscle performance by enhancing hypertrophy. This is associated with increased AKT/mTOR anabolic signalling pathway, muscle protein synthesis, anabolic signalling, activation of SC and positive up-regulation of genes related to extracellular remodelling [80,81]. Other genes stimulated by 1,25(OH)_2_D-VDR complex include those involved in endothelial function by promoting angiogenic action [44,83]. This is achieved by modulating Nitric oxide (NO) synthesis through endothelial NO synthase (NOS) and endothelial NOS (eNOS) activity [44,83]. The regulated production of NO can improve endothelial function by promoting angiogenic activities, increasing blood flow, facilitating the delivery of oxygen and nutrients to the muscles, as well as the elimination of wasting products such as ammonia. An optimal blood flow to muscle tissue will affect an athlete’s recovery speed by regulating blood pressure, improving nutrient distribution, contributing to expand muscle fibres, and increasing cardiovascular endurance. All these NO actions would improve sports performance [84]. Altogether, the direct and indirect effects induced by 1,25(OH)_2_D-VDR complex lead to improvements in muscle health, functionality, strength, recovery and athletic performance [6,75]. Therefore, optimal circulating levels of 25(OH)D are associated with better performance due to the role of vitamin D in muscular strength. These results go in the line of those reported for sarcopenic patients [33,47].

In addition to correct concentration of circulating vitamin D, the expression levels of VDR are important as well [81]. Low expression of VDR is related to muscle pathologies and aging [85]. Decreased VDR expression in muscle substantially impairs myogenic differentiation induced by vitamin D [86]. However, increases in VDR expression together with optimal 25(OH)D levels are related to tissue regeneration after muscle damage [87]. In this line, 25(OH)D plasma concentration ≥ 30 ng/mL induces muscular upregulation of VDR [64,65,81,87]. However, SNP rs2228570 is the only VDR polymorphism that presents structural changes for the VDR protein [48]. Accurate VDR structure is essential for transactivating or transrepressing functions, through interaction with coregulatory nuclear proteins, forming heterodimers that allow positive or negative transcription modulation of target genes [88].

We have identified that VDR genotype variants (rs2228570) present significant differences (*p* < 0.05) in circulating levels of 25(OH)D. Our results showed a negative correlation (r = −0.43; *p* < 0.001) between plasmatic concentration of 25(OH)D and the homozygous GG biallele. Furthermore, CrossFit^®^ athletes carrying the AA biallele were three times more likely (OR 2.88 [14.43–5.92]) to have higher levels of circulating vitamin D. In this context, rs2228570 (*FokI* polymorphism) is the only VDR polymorphism that modifies the length of the protein, conditioning vitamin D action [51]. In addition, deficient (<10 ng/mL) or toxic (>150 mg/mL) 25(OH)D levels inactivate the biologically active form of VDR [51].

The *VDR* gene with the homozygous *FokI* AA biallele results in increased VDR protein levels compared to GA or GG genotypes [89]. Our results can confirm this observation, GG being the biallelic variant that displays the lowest circulating VDR levels. Thus, *VDR* gene polymorphisms may condition VDR expression and protein stability [90]. In this line, *VDR* expression is elevated acutely (1–3 h) after resistance exercise [64]. Although low levels of *VDR* expression in muscle still can allow direct physiological actions, our findings suggest that the A allele is a protective factor for having optimal levels of 25(OH)D [33] and expression of the complete VDR protein leading to optimal biological activity [48]. Therefore, the A allele of *VDR* rs2228570 was associated with increased *VDR* messenger RNA (mRNA) copies [45,91]. In this context, we identified 12 advanced CrossFit^®^ practitioners and 4 of elite level carrying the A allele of *VDR* (rs228570), all (n = 16) included in the competitor level (Table 8).

### 3.1. Limitations and Strengths

Our study has some limitations that may have impacted the findings and should be considered when interpreting our results. Our cohort included a limited number of male-only CrossFit^®^ practitioners, as it is an exploratory or pilot trial, which could limit generalizability and statistical power. The SNPs of three candidate genes were studied, but the influence on 25(OH)D levels could be multigenic. Also, our participants were only from one type of sport, CrossFit^®^, which may not be representative of the overall athlete population, although CrossFit^®^ is a complete exercise with multimodal physiological components. We checked food intake, and the participants did not receive vitamin D supplements, which would not distort the 25-OH/D levels. Additionally, 20% of the total samples were genotyped in duplicate as a quality control test, and all results were consistent for three of the candidate SNPs. Therefore, this could suggest that the analyses and results could be considered honest.

### 3.2. Future Directions

Given all this, the results derived from the study of the SNPs described in this report could allow athletes to modulate plasmatic levels of vitamin D, avoiding fatigue, overtraining, and health problems. In this line, the presented genomic results could allow athletes, coaches, and sports nutritionists to identify individuals at potential risk of low circulating levels of vitamin D, thereby optimising possible interventions. Moreover, the presented data are instrumental for precision personalised nutrition and/or supplementation plans [34], improving health, functionality, and muscle performance in athletes. However, we consider that it is necessary to complement this information considering other characteristics of practitioners in future research such as gender, age, anthropometric composition, health status, sports levels, intensity, and length of exercise, together with dietary preferences and food intolerances or allergies.

## 4. Material and Methods

### 4.1. Study Design

A multicentre observational, longitudinal, pilot study was conducted in 2 CrossFit^®^ Boxes. Results are reported according to the Strengthening the Reporting of Observational Studies in Epidemiology (STROBE) statement [92]. Figure 1 includes a consort diagram outlining the exclusion criteria. The sample involved highly trained male CrossFit^®^ athletes (n = 50) from 2 sports centres located in the cities of Salamanca and Soria (Spain). The study was approved by the Clinical Research Ethics Committee (CREC) of Valladolid Clinical Hospital (PI-19-1350) (Spain). All subjects provided written informed consent, in accordance with the Declaration of Helsinki and the 2013 Fortaleza revision [93]. The sample size was determined using G*Power 3.1 software [94]. A power analysis (1-β error probability) determined that a sample size of 50 participants was sufficient to detect a difference of at least 1% in the serum 25(OH)D concentrations. The power was set at 0.95, and the effect size was estimated to be 0.8 [95].

### 4.2. Inclusion Criteria

The cohort (n = 50) consisted of highly trained CrossFit^®^ athletes over 18 years of age. Practitioners met the following inclusion criteria: (i) ≥20 months of experience training CrossFit^®^; (ii) ≥2 participations in CrossFit^®^ competitions in the last season; (iii) completed Fran WODs < 250 s; (iv) passed a pre-study medical examination to rule out pre-existing illnesses or injuries; (v) did not use illegal drugs according to the World Anti-Doping Agency (WADA) [96] (stimulants, blood derivatives, anabolic steroids) or take medications (i.e., tramadol) or other ergogenic products, including vitamin D supplementation, which could modify muscle function and disturb sports performance tests; (vi) were informed about all possible risks or discomforts and benefits associated with the study and signed the consent form.

### 4.3. Data Collection

Two study investigators collected specific data prior intervention, including sociodemographic information, anthropometric parameters, physical performance level and nutritional habits, and statistically analysed (Appendix A). Gender, age, ethnicity, body mass, fat mass, free fat mass, and height were included as sociodemographic and anthropometric characteristics. Body mass, fat mass, and free fat mass were assessed by a single observer through resistance and reactance measurements obtained with a bioimpedance analyser (BC-730; Tanita^®^, Tokyo, Japan). A constant alternating current of 800 µA and a frequency of 50 kHz was used [31]. Bioelectrical bioimpedance was used because it has been considered an accurate method to evaluate anthropometric parameters [97]. Height was determined with a stadiometer.

Physical performance was evaluated regarding maximum oxygen consumption (VO_2_max) determined by a modified Bruce treadmill protocol [98]. Individuals with optimal status passed to Fran WODs in order to participate in the study. Fran WODs include 3 rounds of stablished thrusters and pull-ups for 21, 15, and 9 repetitions [13]. This test was carried out to ensure a minimal physical condition of the CrossFit^®^ participants in the study. Fran is one of the most famous types of WODs that all CrossFit^®^ practitioners use to monitor performance improvements, measure physical capacity, and analyse progress [99]. A time of 250 s is the cut-off point for participation in local amateur CrossFit^®^ competitions, and reaching this achievement is considered an advanced CrossFit^®^ level [55]. In this line, 250 s in Fran execution was considered as the main inclusion criteria in the study. On the other hand, CrossFit^®^ Total was considered to evaluate the performance level of participants during intervention. In CrossFit^®^ Total, participants must complete one RM of shoulder press, back squat, and deadlift in 90 min under supervision. Participants were allowed to perform 3 attempts to achieve RM, with 180 to 300 s of rest between each. A summation of individual RM loads (in Kg) was performed to determine a total score that indicates level of each participant [12,13]: (i) <270 Kg beginner; (ii) 271–360 Kg intermediate; (iii) 361–450 Kg advanced; (iv) ≥451 Kg elite. For participation in local amateur CrossFit^®^ competitions, ≥360 kg is the cut-off point in CrossFit^®^ Total [55]. The training period was the same for all participants and included 4 weekly sessions on alternate days (Monday, Wednesday, Friday, and Saturday). Each session lasted 80 min and was divided into a CrossFit^®^ warm-up, CrossFit^®^ strength and/or skill technique, structured strength, or conditioning workout (50–60 min) followed by stretching and/or cool-down. All training was planned and supervised by a certified CrossFit^®^ trainer with a grade I or II certificate.

Finally, nutritional evaluation was carried out following previous published protocols by our group [31,98]. CrossFit^®^ athletes were briefed on the Food Frequency Questionnaire (FFQ) and completed it with the assistance of a study investigator who recorded the subjects’ daily food and fluid intake. The values of each food were transformed into micronutrients, macronutrients, and total energy consumption using the validated Easy Diet© software 7.2 (https://www.easydiet.es/ accessed on 10 February 2024). In addition, the total energy intake/Kg for each athlete was assessed [100,101].

### 4.4. Blood Collection and 25(OH)D Determination in Peripheral Blood

25(OH)D concentration is the best indicator of vitamin D status because 25(OH)D has a longer half-life (2–3 weeks) than 1,25(OH)_2_D (4 h) [69]. Also, the circulating concentration of 1,25(OH)_2_D or calcitriol is modulated by parathyroid hormone (PTH), and is sensitive to serum calcium and phosphorus concentrations. In addition, circulating 1,25(OH)_2_D does not correlate with tissue vitamin D stores [102]. 1,25(OH)_2_D concentration may be above or within the normal clinical range when vitamin D stores are deficient [102]. Circulating 25(OH)D levels were determined as indicated previously [33].

All blood samples were collected under basal and fasting conditions, with a fasting period of at least 12 h after the last meal. All blood samples were taken at 8:30 am, and all participants were comfortably seated or lying down. Participants were called to the laboratory at 8:30 am. The Vacutainer system was used (10 mL for serum and 5 mL and 3 mL for EDTA). Immediately after collection, the tubes were inverted 10 times and placed in a sealed box for storage at 4 °C. Temperature during transport was monitored using a specific label (Libero Ti1, Elpro, Buchs, Switzerland), which was used to measure and record the temperature. Samples were transported under appropriate conditions and arrived at the laboratory 30 min after extraction. Delays did not affect the analytical quality of the parameters studied. Samples containing EDTA (anticoagulant) were homogenised for 15 min before analysis [100]. Plasma obtained in EDTA tubes was assayed for 25(OH)D concentration using a commercially available ELISA (Enzyme-Linked Immunosorbent Assay) kit (Eagle Biosciences, Nashua, NH, USA). 25(OH)D is the established biomarker of vitamin D (1,25(OH)D) status [101]. Reference circulating levels of 25(OH)D for athletes were established according to the limits in blood defined by researchers [6,102,103,104,105] of vitamin D as: [6]: (i) deficiency (<20 ng/mL); (ii) insufficient (<30–32 ng/mL); (iii) sufficient (>30–32 ng/mL); (iv) optimum (40–100 ng/mL); (v) hypercalcemia toxic (>150 ng/mL).

### 4.5. Single-Nucleotide Polymorphism (SNPs) Selection

We selected 3 genes related to vitamin D synthesis (*CYP2R1*), transport (*GC*), and function as vitamin D receptor (*VDR*). The SNPs of the genes were: rs10741657 to *CYP2R1*, rs2282679 to *GC*, and rs2228570 to *VDR*. SNPs had a function already described in the literature based on their previous association with 25(OH)D and its related genotypes [33]. The *CYP2R1* gene has a frequency in the Caucasian adult population of 60–65% [58]. The allele rs2282679 for *GC* gene has a frequency in the Caucasian adult population of 30–40% [106]. Finally, the frequency of allele rs2228570 for the *VDR* gene in the adult Caucasian population is 39% [107]. The frequency of these alleles is generally consistent across different Caucasian populations and does not differ markedly between genders.

### 4.6. Single-Nucleotide Polymorphism (SNPs) Determination After DNA Isolation and Genotyping

We evaluated 3 SNPs in 3 different genes related to vitamin D metabolism, transport, and function: rs10741657 to *CYP2R1*, rs2282679 to *GC* and rs2228570 to *VDR*. These SNPs display an association with 25(OH)D circulating levels as described previously [40,45]. Isolation and genotyping of DNA was carried out following the methodology previously described [33]. The blood sample was placed in a BD Vacutainer^®^ ETDA test tube and stored in a refrigerated space in the gym. Then, tubes were transported in cold containers to the laboratory to perform DNA extraction. Each test tube was labelled with a unique identification number that correlated with a code in the patient’s individual consent form to keep patient information confidential throughout the data collection process. Genomic DNA was isolated from 20 mL of the blood sample using FlexiGene DNA Kit (Qiagen, Hilden, Germany). NanoDrop ND-1000, version 3.3 (ThermoFisher Scientific, Waltham, MA, USA) was used for DNA quantification. The 3 SNPs were genotyped with KASPar assays (KBiosciences, Herts and LGC Genomics, Hoddesdon, UK) according to the manufacturer’s instructions. The KASPar assay system is based on the discriminatory power of a new form of allele-specific competitive PCR to determine alleles at a specific locus within genomic DNA for SNP typing. SNP to assay conversion rate > 90%, error rate and reproducibility should be < 0.3% [108]. The sample success rate was 96.1%. SNP calling rates (successfully genotyped samples) ranged from 96.7 to 99.8% (median, 99%). In general, 20% of the samples were randomly selected to be genotyped in duplicate as a quality control. Agreement rates between blinded replications were 100%. Previous studies [46,59] that have used this genotyping technology have found that 10% of samples were genotyped in duplicate as a quality control.

### 4.7. Assessment of Genotyping and Phenotyping Frequencies

To calculate the frequency of each genotype in the study population, the number of individuals with a given genotype was divided by the total number of individuals in this population. Thus, D is the frequency of homozygotes for one allele (A1), R the frequency of homozygotes for the other allele (A2), and H the frequency of heterozygotes. Therefore, the addition D + R + H = 1. To calculate the frequency of each allele in the study population, the number of copies of each allele (A1 and A2) has to be divided by the total number of copies of all the alleles existing in the population for the locus considered. Thus, if p is the frequency of allele A1 and call q the frequency of allele A2: p = D + 1/2H; q = R + 1/2H. The global rs10741657 to *CYP2R1*, rs2282679 to *GC*, and rs2228570 to *VDR* allele frequencies were drawn using the GnomAD database [109]. Hardy–Weinberg equilibrium (HWE) principles were applied to detect genotyping error [110].

### 4.8. Determination of Serum Protein Levels for Vitamin D–Binding and Vitamin D Receptor

Plasma samples were vortexed and subsequently centrifuged (14,000 rpm). The supernatant was used for measurements. Human circulating VDBP was measured with a commercially available sandwich ELISA (Immundiagnostik catalog # K2314, Bensheim, Germany) according to the manufacturer’s instructions. Briefly, plasma (diluted 1:40,000) was incubated in a microtiter plate coated with polyclonal anti-VDBP antibodies for one hour. Subsequently, a polyclonal peroxidase-labelled rabbit-anti-VDBP detection antibody was added and incubated for one hour. After washing, tetramethylbenzidine was added as a substrate for 15 min. After adding a stop solution, absorbance at 450 nm was measured by a Microplate Photometer Biosan (Bonsai Lab S.L., Madrid, Spain). The obtained VDBP levels of plasma and serum samples have to be multiplied with a dilution factor of 40,000. Using a standard curve generated with VDBP protein as provided by the manufacturer, final VDBP concentrations were calculated. The detection limit of this ELISA is 1.23 ng/mL; intra-assay CV < 5.0% for 16 replicate determinations at concentrations of 24.2 and 42.9 mg/dl and inter-assay CV < 12.7% for a concentration of 19.3 mg/dl in 14 different assays on two different lots; recovery ranges from 85 to 116%, and linearity was acceptable (r^2^ = 0.998) [111].

Plasma VDR concentration was determined by using the “human vitamin D receptor ELISA” kit (SinoGeneClon Biotech Co. Ltd., Hangzhou, China), according to manufacturer instructions. Each sample was determined in duplicate. Detection range of the kit is 0.1–8 ng/mL, with a sensitivity of 0.05 ng/mL [112].

### 4.9. Data Management and Statistical Analysis

Means and standard deviations (SD) were calculated for continuous variables, while frequencies and percentages were used for categorical variables. To assess the relationship between single-nucleotide polymorphisms (SNPs) and circulating 25(OH)D levels, a univariate linear regression model was applied, considering the genetic variable as a fixed factor. A Bonferroni post hoc test was then performed to determine significant differences between SNPs. Correlations between SNPs and 25(OH)D levels were estimated using Spearman’s rank correlation coefficient. To investigate the association between bi-allelic variants of each SNP and 25(OH)D concentration (<30 ng/mL *vs*. ≥30 ng/mL, classified as deficient or sufficient for athletes), multivariate logistic regression models were conducted, calculating odds ratios (OR) with their corresponding 95% confidence intervals (CI). Adjusted models accounted for body mass index (BMI), maximal oxygen consumption (VO_2_max), and age. The HWE principle was applied to detect potential genotyping errors. Statistical significance was set at *p* < 0.05 for all analyses. Statistical procedures were performed using STATA version 15 (STATA Corp., College Station, TX, USA). Additional details on statistical assumptions and supplementary tests are available in the supplement.

## 5. Conclusions

The present study demonstrates that allelic variations in the *CYP2R1* (rs10741657), *GC* (rs2282679), and *VDR* (rs2228570) SNPs affect the vitamin D sufficiency status in CrossFit^®^ athletes. The AA (rs10741657 and rs2228570) and TT (rs2282679) bialleles were 2–3 times more likely to present higher levels of vitamin D. Therefore, genetic determinants can condition 25(OH)D blood levels modulating physiological function and gene expression related to skeletal muscle performance and/or health. In this context, our study indicates that vitamin D circulating levels have a moderate positive correlation (r = 0.41) with total CrossFit^®^ scores in the studied athletes. Therefore, genetic polymorphisms in vitamin D-pathway related to vitamin metabolism (*CYP2R1*, rs10741657), transport to muscle tissue (*GC*, rs2282679) and muscle gene expression (*VDR*, rs2228570) should be considered for precise supplementation.

## Figures and Tables

**Table 1:** Sociodemographic information, anthropometric parameters and Fran WOD results of the study participants.

Characteristics	CrossFit^®^ Athletes	
Sample size (n)	50	
Age (years)	35.7 ± 11.3	
Gender n (%)	Male	50 (100)
	Female	0 (0)
Ethnicity n (%)	Caucasian	50 (100)
	Other	0 (0)
Body mass (Kg)	77.6 ± 10.9	
Fat Mass (Kg)	9.7 ± 2.9	
Fat Mass (%)	12.5 ± 2.3	
Fat Free Mass (Kg)	67.9 ± 4.1	
Fat Free Mass (%)	65.2 ± 2.6	
Height (cm)	171.5 ± 5.6	
VO_2_max (mL/kg/min)	43.5 ± 4.4	
Crossfit^®^ experience (months)	35.3 ± 11.7	
Fran WODs * (seconds)	231 ± 15	

Data are expressed as mean ± standard deviation (SD). Abbreviations: WODs: Workouts of the day; VO_2_max: maximum oxygen consumption. (*) Three rounds of thrusters and pull-ups for 21, 15, and 9 repetitions.

**Table 2:** Daily energy and micronutrient intake in CrossFit^®^ participants.

CrossFit^®^ participants (n)	50
Energy (kcal/kg)	40.3 ± 4.8
Proteins (g)	141.3 ± 37.9
Fats (g)	134.3 ± 43.2
Carbohydrates (g)	341.2 ± 97.6
Ca (mg)	1026.3 ± 224.1
Mg (mg)	544.3 ± 97.2
P (mg)	2120.6 ± 67.1
Fe (mg)	23.1 ± 3.6
Zn (mg)	13.4 ± 1.1
Vitamin A (µg RE)	1862.3 ± 1177.1
Vitamin E (mg)	16.0 ± 1.8
Vitamin B_1_ (mg)	2.9 ± 0.4
Vitamin B_2_ (mg)	2.6 ± 0.3
Vitamin B_3_ (mg NE)	40.9 ± 6.1
Vitamin B_6_ (mg)	4.3 ± 0.5
Vitamin B_9_ (mg)	637.2 ± 172.1
Vitamin B_12_ (µg)	9.6 ± 2.7
Vitamin C (µg)	351.1 ± 140.2

Data are expressed as mean ± standard deviation (SD). Abbreviations used: NE, niacin equivalents; RE, retinol equivalents.

**Table 3:** Circulating levels of 25-hydroxy vitamin D (25(OH)D) and characterisation by ranges of deficiency, sufficiency, optimum and toxicity.

CrossFit Competitive Categories (Years)	Sample (n)	25-(OH)D (ng/mL)	Deficiency n (%) <20 ng/mL	Insufficiency n (%) <30–32 ng/mL	Sufficiency n (%) >30–32 ng/mL	Optimum n (%) 40–100 ng/mL	Toxic n (%) >150 ng/mL
Absolute/Elite 18–35	19	36.2 ± 4.3	-	2 (10.5)	14 (73.7)	3 (15.8)	-
Master >35	31	33.1 ± 6.8	-	6 (19.3)	20 (64.5)	5 (16.2)	-
Elite + Master	50	34.7 ± 5.2	-	8 (16.0)	34 (68.0)	8 (16.0)	-

Data are expressed as mean ± standard deviation (SD) for quantitative variables and as frequency (%) for categorical variables. Characterisation of 25(OH)D levels and ranges in athletes’ populations according to categories of Competition Rulebook CrossFit^®^ [55].

**Table 4:** Circulating levels of 25-hydroxy vitamin D (25(OH)D), vitamin D binding protein (VDBP) and vitamin D receptor (VDR) protein, found in participants with the indicated single-nucleotide polymorphisms (SNPs).

Gen	SNPs	Alleles	25(OH)D (ng/mL)	*p*-Value	VDBP (µg/mL)	*p*-Value	VDR (ng/mL)	*p*-Value
*CYP2R1*	rs10741657	AA	38.2 ± 11.2	0.076	323.6 ± 21.2	0.96	3.8 ± 0.5	0.67
		GA	26.9 ± 7.5		318.1 ± 19.1		3.4 ± 0.6	
		GG	21.5 ± 4.7		316. ± 23.5		3.2 ± 0.8	
*GC*	rs2282679	TT	42.6 ± 3.2	<0.05	344.1 ± 14.3	0.05	3.9 ± 0.9	0.19
		GT	25.4 ± 5.7 *		323.6 ± 16.2		3.3 ± 1.1	
		GG	21.6 ± 5.1 *		301.6 ± 10.2 ^#^		3.1 ± 0.7	
*VDR*	rs2228570	AA	35.9 ± 8.3	<0.05	319.6 ± 17.2	1.1	4.2 ± 0.5	0.05
		GA	24.4 ± 5.6		314.6 ± 19.3		3.6 ± 0.8	
		GG	18.9 ± 4.9 ^$^		311.7 ± 26.7		3.0 ± 0.3 ^&^	

Values are expressed as mean + standard deviation (SD) for quantitative variables. Statistically significant values were considered for *p* < 0.05. Multiple comparisons are based on Bonferroni test. (*) Significant differences in circulating 25(OH)D levels respect to TT allele. (^$^) Significant differences in circulating 25(OH)D levels respect to AA allele; (^#^) Significant differences in circulating VDBP levels respect to TT allele; (^&^) Significant differences in circulating VDR levels respect to AA allele.

**Table 5:** Genotypic and allelic frequency of polymorphisms (SNPs) of the CYP2R1, GC and VDR genes present in the study participants.

Gen (SNPs)	n (%) ^a^	Genotypic Freq ^b^	Allelic Freq ^c^	Alleles	Allele Freq ^d^		HWE ^e^
***CYP2R1*** **(rs10741657)**			A (p) 0.59 G (q) 0.41	A > G	Ref A: 0.379	Alt G: 0.614	Yes 0.023
AA	17 (34)	0.34					
GA	25 (50)	0.50					
GG	8 (16)	0.16					
A	59 (59)						
G	41 (41)						
***GC*** **(rs2282679)**			T (p) 0.60 G (q) 0.40	T > G	Ref T: 0.716	Alt G: 0.283	Yes 0.34
TT	19 (38)	0.38					
GT	22 (44)	0.44					
GG	9 (18)	0.18					
T	60 (60)						
G	40 (40)						
***VDR*** **(rs2228570)**			A (p) 0.60 G (q) 0.40	A > G	Ref A: 0.387	Alt G: 0.612	Yes 0.34
AA	21 (42)	0.42					
GA	18 (36)	0.36					
GG	11 (22)	0.22					
A	60 (60)						
G	40 (40)						

Abbreviations: Ref = the allele in the reference genome; Alt = any other allele found at that locus; HWE: Hardy–Weinberg equilibrium; n = sample; Freq = frequency (number of observations/total number). ^a^ Values are expressed as frequency (percentage) for categorical variables. ^b^ Results obtained from Genotypic frequency = number of individuals with the genotype/total number of individuals in the population. ^c^ Assessed by calculating p and q (allelic frequencies) from the genotypic frequency observed in the population p = D + 1/2H; q = R + 1/2H. ^d^ dbSNP: NCBI database of genetic variation https://www.ncbi.nlm.nih.gov/snp/?term= (accessed on 12 november 2024). ^e^ Tests of Hardy–Weinberg equilibrium. HWE was performed using a simple χ^2^ goodness-of-fit test.

**Table 6:** Correlations between the concentration of 25-hydroxy vitamin D and single-nucleotide polymorphisms (SNPs) of CYP2R1, *GC* and *VDR* genes involved in vitamin D function.

Gen (SNPs)	(n = 50)	
	r	*p*
*CYP2R1* (rs10741657)		
AA	0.17 *	0.034
GA	0.089	0.424
GG	−0.34 *	0.016
*GC* (rs2282679)		
TT	0.29 *	0.041
GT	0.07	0.526
GG	−0.33 *	0.012
*VDR* (rs2228570)		
AA	0.15 *	0.030
GA	0.06	0.172
GG	−0.43 *	<0.001

(*) Statistically significant values (*p* < 0.05). Correlations (r) are based on Spearman’s rank correlation coefficient.

**Table 7:** Multivariate logistic regression analysis of participant study characteristics and single-nucleotide polymorphisms associated with 25-hydroxy vitamin D circulating concentrations.

Variable	Full Cohort (n = 50)	
	OR (95% CI) Crude	OR (95% CI) Multivariate ^1^
Body mass index (BMI), (Kg/m^2^)	1.00 (ref)	--
VO_2_ max (mL/Kg/min)	1.62 (0.81–3.27)	1.77 (0.54–3.65)
Age (years)	0.91 (0.71–1.18)	0.92 (0.62–1.46)
*CYP2R1* (rs10741657)	1.00 (ref)	--
AA	1.44 (0.74–2.87)	2.01 (0.77–5.48) *
GA	0.93 (0.782–1.05)	1.02 (0.83–1.27)
GG	0.83 (0.74–0.93)	0.76 (0.65–0.89)
*GC* (rs2282679)	1.00 (ref)	--
TT	3.69 (2.28–5.99)	3.67 (2.11–6.41) *
GT	0.83 (0.68–1.02)	0.76 (0.49–1.21)
GG	0.67 (0.53–0.85)	0.66 (0.51–0.89) *
*VDR* (rs2228570)	1.00 (ref)	--
AA	2.93 (1.58–5.47)	2.88 (1.43–5.92) *
GA	1.01 (0.42–2.64)	1.24 (0.29–6.11)
GG	0.53 (0.23–1.42)	0.31 (0.12–1.27) *

Abbreviations: BMI, body mass index; CI, confidence interval; OR, Odds Ratio; ref, reference. (*) Statistically significant respect to optimal levels of circulating 25(OH)D (*p* < 0.05). (^1^) Multivariate model adjusted for all variables in the table.

**Table 8:** Single-nucleotide polymorphisms (SNPs) of *CYP2R1*, *GC* and *VDR* genes present in study participants according with their CrossFit^®^ level, evaluated through CrossFit^®^ total (see Materials and Methods).

Gen	SNPs	Allele	n (%)	Levels of CrossFit^®^ Total (n) ^1^				
				Beginner (Level 0) <270 Kg	Intermediate (Level 1) 271–360 Kg	Advanced (Level 2) 361–450 Kg	Elite (Level 3) ≥451 Kg	Competitors ^2^ (Levels 2 + 3) ≥360 Kg
*CYP2R1*	rs10741657	AA	17 (34)	0	6	8	3	11
		GA	25 (50)	2	18	4	1	5
		GG	8 (16)	4	3	1	0	1
		AA/GA/GG	50 (100)	6	27	13	4	17
*GC*	rs2282679	TT	19 (38)	1	8	7	3	10
		GT	22 (44)	3	15	4	0	4
		GG	9 (18)	2	4	2	1	3
		TT/GT/GG	50 (100)	6	27	13	4	17
*VDR*	rs2228570	AA	21 (42)	0	11	6	4	10
		GA	18 (36)	2	10	6	0	6
		GG	11 (22)	4	6	1	0	1
		AA/GA/GG	50 (100)	6	27	13	4	17

Values are expressed as frequency (percentage) for categorical variables. Abbreviations = SNPs: Single-nucleotide polymorphisms; %: percentage; kg: kilograms. ^1^ Glassman G. CrossFit training guide level 1 [12]. ^2^ Competition RuleBook CrossFit^®^ Games [55].

## Data Availability

All data supporting the findings of this study are available within the paper.

## Abbreviations

(1,25(OH)_2_D)	1,25-dihydroxy vitamin D
(24,25(OH)_2_D)	24,25-dihydroxy vitamin D
25(OH)D	25-hydroxy-vitamin D
CI	confidence interval
CREC	Clinical Research Ethics Committee
ELISA	Enzyme-Linked Immunosorbent Assay
eNOS	endothelial NOS
FFQ	Food Frequency Questionnaire
GDF-8	myostatin
HIIT	high-intensity interval training
HWE	Hardy–Weinberg Equilibrium
IGF-1	insulin-like growth factor-1
MAF	macrophage activating factor
MAFr	mayor allele frequency
mRNA	messenger RNA
NO	Nitric oxide
NOS	endothelial NO synthase
RED-S	Relative Energy Deficiency in Sport
RM	repetition maximum
SC	satellite cell
SD	standard deviations
SNPs	single-nucleotide polymorphisms
STROBE	Reporting of Observational Studies in Epidemiology
VDBP	vitamin D-binding protein
VDR	vitamin D-receptor
VDRE	vitamin D responsive elements
WADA	World Anti-Doping Agency
WODs	Workouts of the Day

## Appendix A. Statistical Details

### Appendix A.1. Assumptions and Data Validation

- Tests for normality (e.g., Shapiro–Wilk) were conducted for continuous variables to determine the suitability of parametric approaches.
- Multicollinearity between predictor variables was assessed using variance inflation factors (VIF), ensuring independent contributions to the logistic regression model.
- Outliers were detected and evaluated using Cook’s distance to minimise undue influence on regression coefficients.

### Appendix A.2. Hardy–Weinberg Equilibrium (HWE) Testing

- The HWE principle was tested using chi-square goodness-of-fit tests, ensuring that allele frequencies matched expected distributions under random mating conditions.
- SNPs deviating from HWE (p < 0.05) were examined for potential genotyping errors or population structure effects.

### Appendix A.3. Regression Model Specifications

- The logistic regression models were fitted using both crude estimates and adjusted models controlling for BMI, VO_2_max, and age.
- Sensitivity analyses were performed by excluding participants with extreme 25(OH)D values (>100 ng/mL) to assess robustness of findings.
- Interaction terms between SNPs and VO_2_max were explored but did not yield statistically significant results (*p* > 0.05).

### Appendix A.4. Statistical Software and Code Availability

- All statistical analyses were performed using STATA version 15.
- Custom scripts for HWE testing and correlation analyses can be provided upon request for reproducibility purposes.
